# PreDigs: A Database of Context-specific Cell Type Markers and Precise Cell Subtypes for Digestive Cell Annotation

**DOI:** 10.1093/gpbjnl/qzaf066

**Published:** 2025-08-07

**Authors:** Jiayue Meng, Mengyao Han, Yuwei Huang, Liang Li, Yuanhu Ju, Daqing Lv, Xiaoyi Chen, Liyun Yuan, Guoqing Zhang

**Affiliations:** National Genomics Data Center & Bio-Med Big Data Center, Shanghai Institute of Nutrition and Health, Chinese Academy of Sciences, Shanghai 200031, China; Putuo People’s Hospital, Shanghai Key Laboratory of Signaling and Disease Research, School of Life Sciences and Technology, Tongji University, Shanghai 200092, China; National Genomics Data Center & Bio-Med Big Data Center, Shanghai Institute of Nutrition and Health, Chinese Academy of Sciences, Shanghai 200031, China; National Genomics Data Center & Bio-Med Big Data Center, Shanghai Institute of Nutrition and Health, Chinese Academy of Sciences, Shanghai 200031, China; Shanghai Southgene Technology Co., Ltd., Shanghai 201203, China; Shanghai Southgene Technology Co., Ltd., Shanghai 201203, China; Ningbo Institute of Life and Health Industry, University of Chinese Academy of Sciences, Ningbo 315000, China; National Genomics Data Center & Bio-Med Big Data Center, Shanghai Institute of Nutrition and Health, Chinese Academy of Sciences, Shanghai 200031, China; National Genomics Data Center & Bio-Med Big Data Center, Shanghai Institute of Nutrition and Health, Chinese Academy of Sciences, Shanghai 200031, China; Shanghai Sixth People’s Hospital, Shanghai 200233, China

**Keywords:** Data integration, Cell type marker, Gastrointestinal tumor, Single-cell RNA sequencing, Database

## Abstract

Research on cell type markers helps investigators explore the diverse cellular composition of gastrointestinal tumors, thereby enhancing our understanding of tumor heterogeneity and its impact on disease progression and treatment response. However, the integration of large-scale datasets and the standardization of cell type identification remain challenging. Here, we developed PreDigs, a user-friendly database of predicted signatures for the digestive system, which offers 124 curated single-cell RNA sequencing datasets, covering over 3.4 million cells, all available for download. After unsupervised clustering, we unified the identification and nomenclature of cell subtype labels, constructing a cell ontology tree with 142 cell types across 8 hierarchical levels. Meanwhile, we calculated three different context-specific cell type markers, including “Cell Markers”, “Subtype Markers”, and “TPN Markers”, based on various application requirements within or across tissues. Through the integrated analysis of PreDigs data, we identified distinct cell subpopulations exclusive to tumors, one of which corresponds to tumor-specific endothelial cells. Additionally, PreDigs offers online cell annotation tools, allowing users to classify single cells with greater flexibility. PreDigs is accessible at https://www.biosino.org/predigs/.

## Graphical abstract

**Graphical Abstract:**
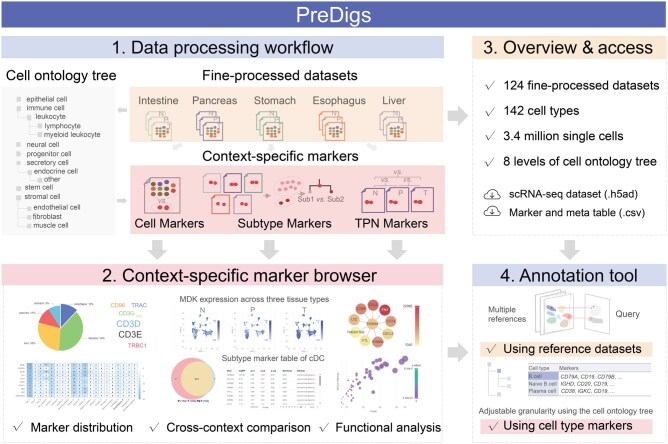

## Introduction

Digestive cancers, including esophageal, gastric, liver, pancreatic, and colorectal cancers, account for more than 50% of global cancer-related morbidity and mortality [1]. As solid tumors, the complex cellular components, including malignant cells, tissue-resident cells, and tumor-infiltrating cells in the tumor microenvironment (TME), influence tumor cell growth and development [2,3]. Leveraging massive single-cell RNA sequencing (scRNA-seq) data enables comprehensive profiling of gene expression at the single-cell level, helping researchers uncover cellular diversity [4], distinguish cell lineages [5], and characterize the TME of digestive cancers [6]. Accurate cell type classification is crucial for single-cell analysis in cancer research and serves as the key to understanding intratumor heterogeneity [7–10].

Until now, great efforts have been made to develop several cell type marker data portals and resources for single-cell classification [11–20]. Notable databases include Cell Ontology (CL) [11], CellMarker [13], PanglaoDB [15], Cell Taxonomy [19], and CellSTAR [20]. CL, a widely used framework for representing cell types, defines hierarchical relationships for approximately 2600 cell types, although most lack associated, assessable cell markers. CellMarker, the first database to compile a curated collection of 27,166 cell markers for 3123 cell types in human and mouse, does not include single-cell transcriptome profiles of these markers. In contrast, databases such as PanglaoDB have compiled cell types, markers, and scRNA-seq datasets; however, inconsistencies in dataset pre-processing procedures and the lack of a unified nomenclature for cell types, such as the adoption of the CL standardization, hinder direct cross-dataset integration and comparison. Cell Taxonomy and CellSTAR have addressed these issues, yet they do not support cross-tissue comparisons, such as analyzing gene expression variations in the same cell type between normal and tumor tissues. This gap limits our understanding of cell type markers in the TME and hinders precise annotation of TME cell types [21].

Here, we presented PreDigs, a comprehensive database of predicted signatures for the digestive system, which integrates extensive scRNA-seq datasets and utilizes sophisticated reference data for cell subtype annotation. PreDigs constructs a detailed cell ontology tree with 142 distinct cell types across 8 hierarchical layers. Each node within the hierarchy is linked to relevant expression profiles, enabling comparative analyses across diverse tissues and datasets. Furthermore, PreDigs affords three types of markers tailored for different research contexts: direct comparison of differentially expressed gene (DEG) profiles for a cell type across tissue types (“Cell Markers”), delineation of gene expression differences within cell subtypes via cross-tissue single-cell analysis (“Subtype Markers”), and identification of cell types specifically distributed within a tumor tissue and their gene functions in Kyoto Encyclopedia of Genes and Genomes (KEGG) pathways (“TPN Markers”). The PreDigs interface allows users to retrieve cell type markers and scRNA-seq datasets for in-depth gene expression and functional enrichment analyses. Additionally, an online annotation tool is available to streamline single-cell classification. It supports annotation using both classical markers and customized scRNA-seq reference datasets, improving classification flexibility and reliability.

## Database content and usage

### Overview of data management and integration in PreDigs

**Figure 1:**
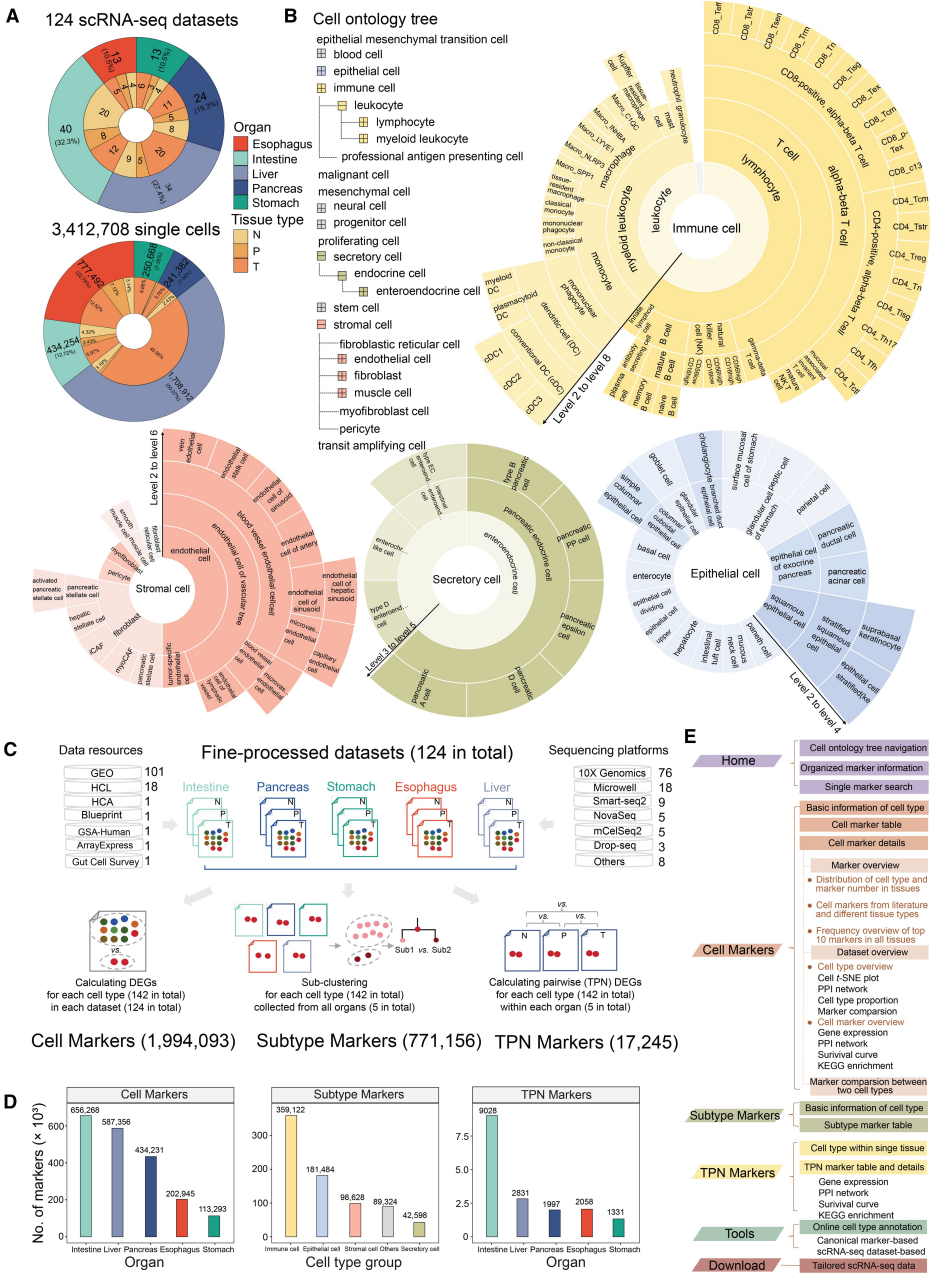
The summary of PreDigs data **A**. PreDigs includes 124 tailored scRNA-seq datasets with 142 cell types, covering nearly 3.4 million cells across 5 digestive organs and 3 tissue types. The pie plots show the distributions of datasets (upper) and single cells (lower) across different digestive organs and tissue types. **B**. An illustration of the cell ontology tree with 8 levels. The cell type names in the hierarchical tree follow the standardized nomenclature and formatting provided by the Cell Ontology database [11]. Except for special cases, all characters are in lowercase, with words separated by spaces. **C**. Overview of data processing workflow for context-specific markers in PreDigs. **D**. Statistics on the number of three types of context-specific markers across different digestive tissues. **E**. Schematic overview of the PreDigs database interface. scRNA-seq, single-cell RNA sequencing; N, normal tissue; P, paracancerous tissue; T, tumor tissue; GEO, Gene Expression Omnibus; HCL, Human Cell Landscape; HCA, Human Cell Atlas; GSA-Human, Genome Sequence Archive for Human; PPI, protein–protein interaction; KEGG, Kyoto Encyclopedia of Genes and Genomes; TPN, tumor–paracancerous–normal pairwise comparison; DEG, differentially expressed gene.

In summary, we obtained 124 well-processed scRNA-seq datasets from adult digestive tissues (see Method; Figure S1). These datasets cover three types of digestive tissues (normal tissue, precancerous tissue, and tumor tissue) across five digestive organs (intestine, pancreas, esophagus, liver, and stomach), comprising approximately 3.4 million single cells (**Figure 1**A). Furthermore, to systematically classify digestive cell types, we developed a hierarchical cell ontology tree with up to 8 levels, incorporating 142 cell types based on the CL database (see Method; Figure S1). Of these, 74% (105 types) correspond to standardized CL identifiers (IDs). The first layer of the cell ontology tree consists of 11 major groups (Figure 1B), with immune cells, stromal cells, secretory cells, and epithelial cells being the 4 primary categories. For instance, as shown in Figure 1B, immune cells are categorized into two main lineages, including lymphocyte and myeloid leukocyte. These are further divided into 64 hierarchical subtypes. PreDigs enhances cell ontology annotation by incorporating 3693 canonical markers across 74 cell types, curated from a comprehensive review. This integration enriches marker-based cell type characterization.

After obtaining the well-processed scRNA-seq datasets and cell ontology tree, we identified three context-specific types of cell markers using different strategies for different applications (see Method; Figure 1C). First, we screened DEGs with significantly higher expression in specific cell types compared to others within each dataset (“Cell Markers”). We then compared DEG sets from the same cell type across different tissue types to identify shared and unique features. Second, we performed cross-tissue integrative analysis to investigate gene expression differences among subtypes within over 140 cell types (“Subtype Markers”). Finally, we integrated multiple datasets within specific tissues to analyze expression differences of specific cell types across three tissue types and performed functional enrichment analysis (“TPN Markers”).

Through this workflow, we obtained 2,782,494 marker records (Figure 1D), including 1,994,093 “Cell Markers”, 17,245 “TPN Markers”, and 771,156 “Subtype Markers”. The number of “Cell Markers” in the intestine, liver, and pancreas was significantly higher than that in the esophagus and stomach. This trend correlates with the number of datasets. However, the number of “TPN Markers” in the intestine is significantly higher than that in other gastrointestinal organs. This could be attributed to the greater variability in TPN among intestinal samples than in other tissues (Figure 1D).

### Web interface and usage

Here, we developed PreDigs, a web resource for viewing cell type markers based on different contextual needs, enabling browsing and comparison across organs, tissues, and cell types as well as downloading tailored scRNA-seq datasets. It includes six key pages (Figure 1E): Home, Cell Markers, Subtype Markers, TPN Markers, Tools, and Download. The Home page offers a cell ontology tree, a summary of data, and marker preparation details. On the three marker pages, a marker table with detailed links to single gene expression and KEGG pathway enrichment results is provided. This setup enables users to explore gene expression features and functional implications after obtaining the marker list. Finally, these data are integrated into an online tool for accurate annotation of digestive cells.

#### Interface for browsing three types of cell markers

**Figure 2:**
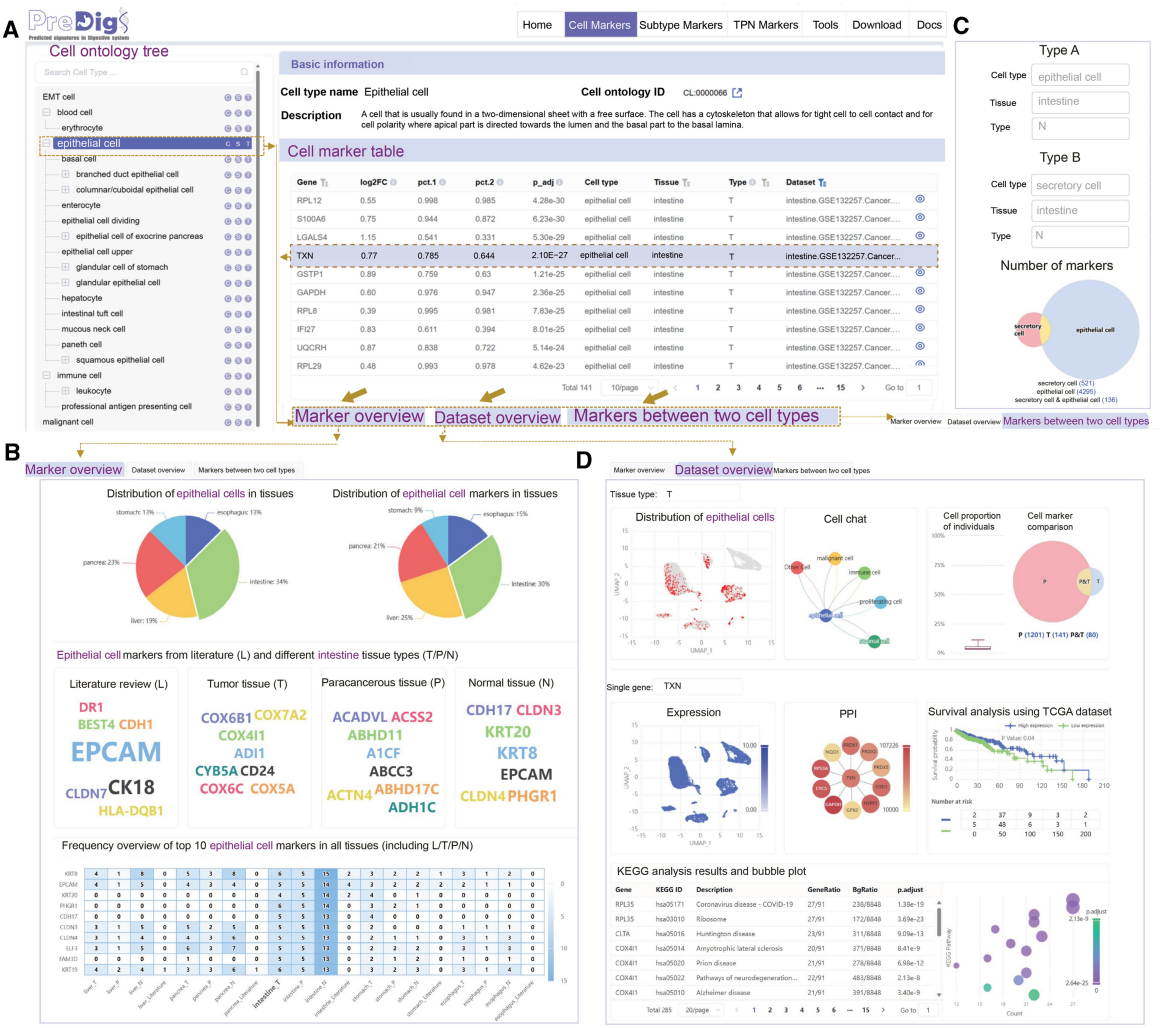
Screenshots of “Cell Markers” interface in PreDigs **A**. “Cell Markers” interface includes a cell ontology tree, a marker table, and three tabs for different functions. **B**. “Marker overview” tab for cell marker statistics. **C**. “Markers between two cell types” tab for comparative analysis. **D**. “Dataset overview” tab for expression profiles of scRNA-seq datasets.

In PreDigs, a cell ontology tree is always displayed on the left side of all web pages, allowing users to browse and select cell types. Three context-specific markers, namely “Cell Markers”, “Subtype Markers”, and “TPN Markers”, are displayed, respectively, across organs, tissues, and cell types. Once a cell type is selected in the cell ontology tree on the left, detailed information including cell type name, cell ontology ID, and description will be displayed first. Meanwhile, the marker list will appear in the table below (**Figure 2**A), where users can click the eye icon on the right to access details for each marker gene. Here, we take “Cell Markers” page as an example to introduce how to browse marker information, while the content on the other two marker pages is relatively simpler but follows a similar structure.

The “Marker overview” tab on the “Cell Markers” page, as shown in Figure 2B, presents an overview of the “Cell Markers” contained within the selected cell type, including their quantitative distribution across gastrointestinal tissues and comparisons among different tissue types. We additionally quantified the occurrences of markers in each tissue and visualized the top 10 most frequently occurring markers, helping users better understand their expression characteristics across different tissues.

The “Markers between two cell types” tab (Figure 2C) facilitates quick comparison between two distinct cell types within a particular tissue, allowing users to instantly view similarities and differences in their marker lists.

The “Dataset overview” tab (Figure 2D) focuses on individual scRNA-seq datasets, enabling users to explore cell clustering via *t*-distributed stochastic neighbor embedding (*t*-SNE), intercellular communication via CellChat, overlapping and unique markers visualized using Venn diagrams, gene expression patterns using *t*-SNE, gene interactions via protein–protein interaction (PPI) networks, prognostic impact through survival analysis, and functional insights through KEGG pathway analysis.

The “Subtype Markers” page provides a research platform for in-depth analysis of subtypes within a given cell type, as defined in the cell ontology tree on the left (Figure S2). We performed refined clustering of each cell type, allowing users to explore the distribution of subtypes across organs and tissue types. This enables a better understanding of subtype diversity and heterogeneity in digestive tissues. Additionally, users can browse detailed markers of each subtype in the table below.

The “TPN Markers” page focuses on identifying DEGs within the same cell type through pairwise comparison across distinct tissue types, namely tumor, paracancerous, and normal tissues. It displays the distribution of the selected cell type within the organ, and across each individual sample (Figure S3). All pairwise comparison markers are presented in the table below, with details for each TPN marker gene accessible via the eye icon on the right. Detailed information, including gene pression [Uniform Manifold Approximation and Projection (UMAP)], gene network (PPI), functional analysis (survival analysis and KEGG enrichment), is displayed. These analytical results help researchers identify key signaling pathways and elucidate their biological significance for further studies.

#### Online cell type annotation platform

**Figure 3:**
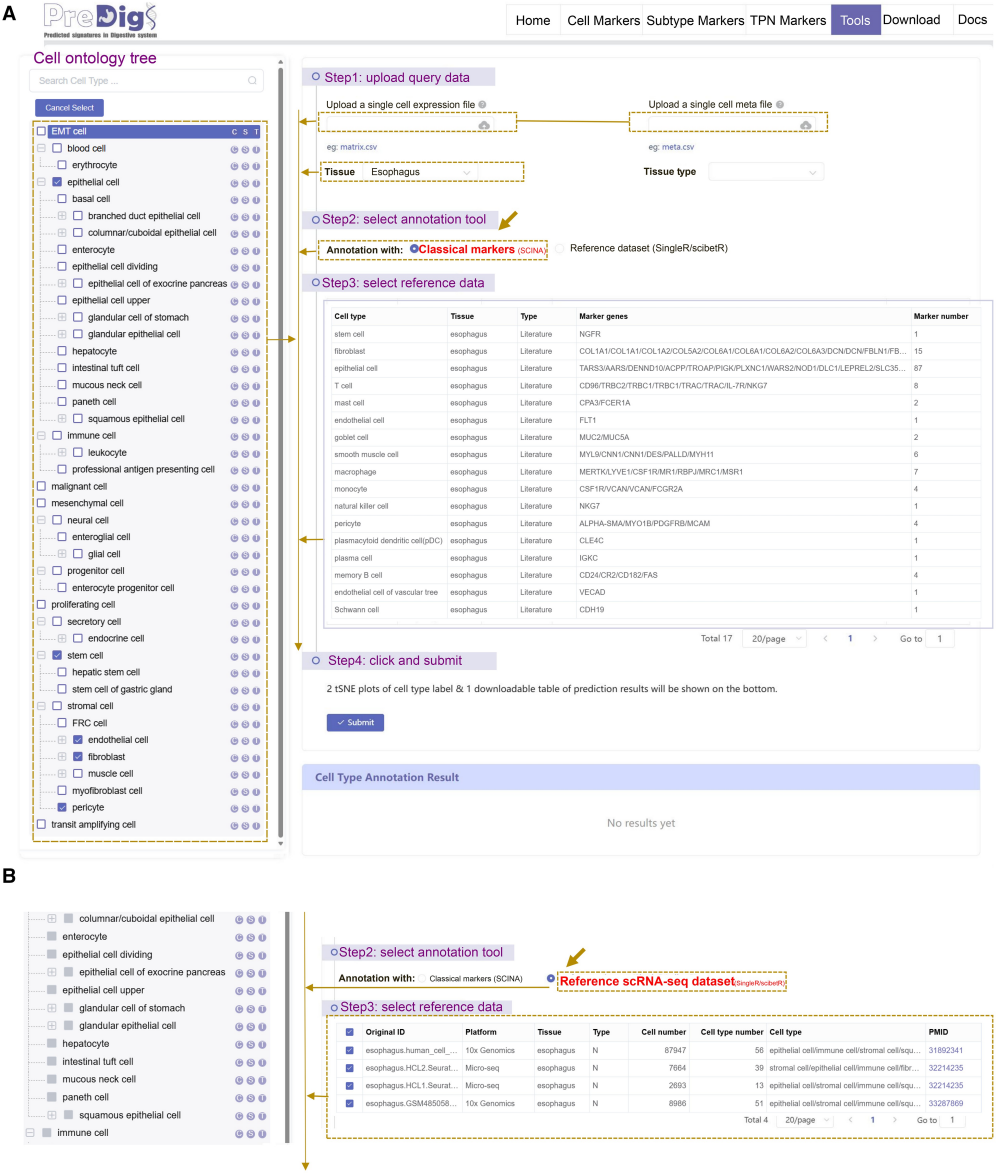
Screenshots of “Tools” interface in PreDigs **A**. Step-by-step instructions for annotation using classical markers as reference data. **B**. Step-by-step instructions for annotation utilizing single or multiple scRNA-seq datasets as reference data.

PreDigs provides an interactive platform for cell type annotation on the “Tools” page (**Figure 3**), offering two annotation methods: cell marker-based annotation and reference dataset-based annotation. Users can upload single-cell expression matrices and metadata tables, and customize their annotation process by selecting automated annotation tools, multiple reference datasets, and additional parameter options. For cell marker-based annotation, users receive a comprehensive list of marker genes for all cell types associated with the selected tissue, with the option to exclude any unnecessary types. For tailored reference datasets, users can select one or more datasets as references for annotation. Upon submission, the platform generates *t*-SNE plots displaying cell group names and predicted labels. Additionally, users can downland prediction results and customized feature gene lists, enhancing both accessibility and efficiency when working with the PreDigs database.

#### Utility of PreDigs reference for revealing cell type heterogeneity

**Figure 4:**
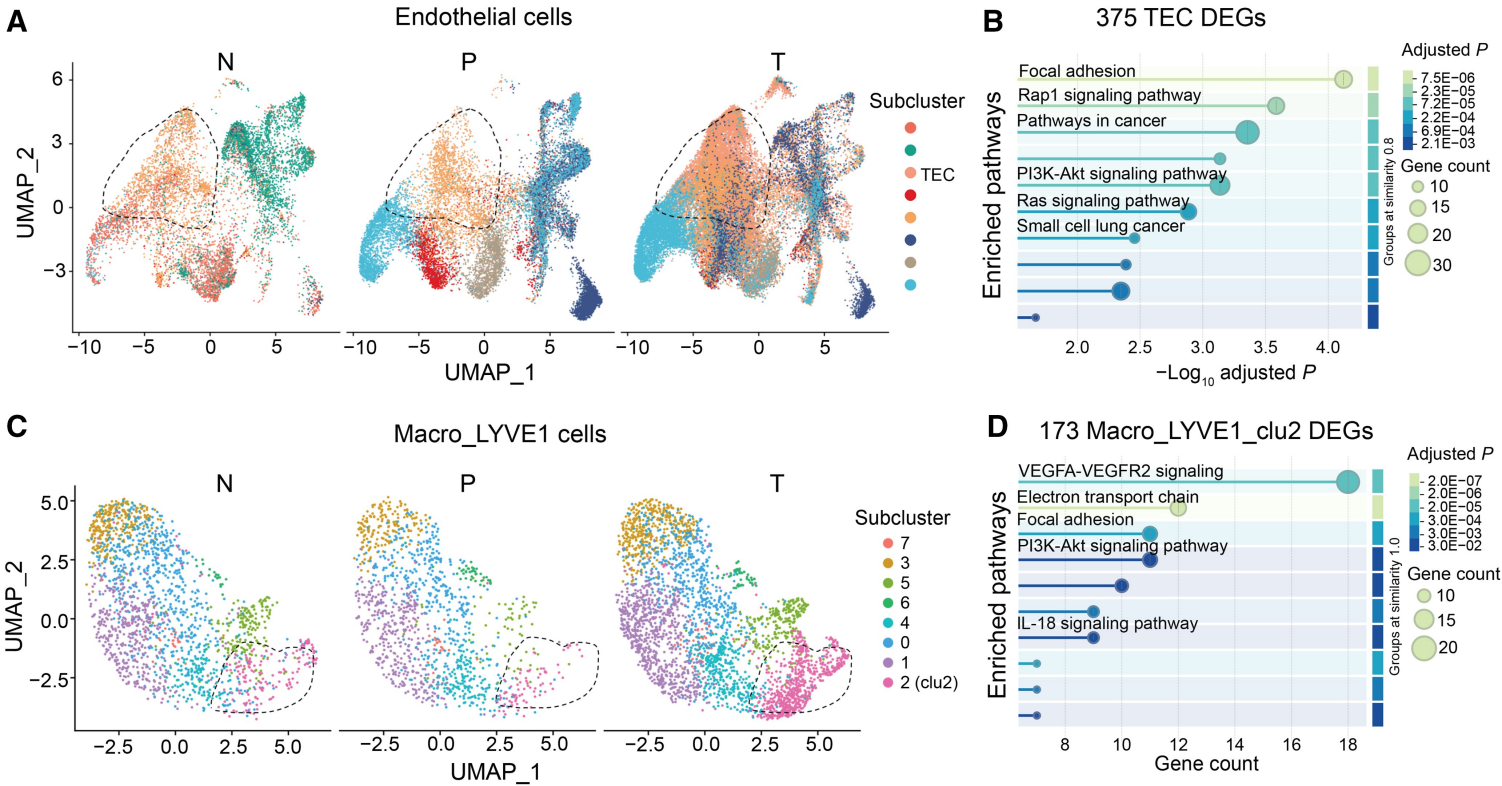
The utility of PreDigs reference for cell subtype annotation **A**. Unsupervised clustering results of endothelial cells from the cross-tissue integration of PreDigs data. **B**. KEGG enrichment results of 375 “Subtype Markers” up-regulated in TECs compared to other endothelial subtypes, using a threshold of adjusted *P* < 1E−04 and log_2_ fold change > 1.5. **C**. Unsupervised clustering results of Macro_LYVE1 cells resulting from the cross-tissue integration of PreDigs data. **D**. Wiki pathway enrichment analysis for 173 “Subtype Markers” up-regulated in clu2 relative to other subclusters within the Macro_LYVE1 cell population, using a threshold of adjusted *P* < 1E−04 and log_2_ fold change > 1.5. TEC, tumor-specific endothelial cell; clu2, cluster 2.

In this study, we first used endothelial cells as an example and performed a large-scale integration of 124 scRNA-seq datasets from 5 digestive organs in the PreDigs database. The integration categorized 96,429 cells into 8 distinct subtypes (**Figure 4**A). Notably, we identified a subtype of 20,019 single cells, predominantly in tumor tissues, designated as gastrointestinal tumor-specific endothelial cells (TECs). Furthermore, we identified 375 “Subtype Markers” highly expressed in TECs compared to other endothelial subtypes. KEGG functional enrichment results revealed that half of the top 10 enriched pathways for these markers were cancer-related, including “Pathway in cancer”, “PI3K-Akt signaling pathway”, and “Small cell lung cancer”, highlighting the pathological state of endothelial cells in tumor tissue (Figure 4B).

Using a similar approach, we integrated and clustered 6346 Macro_LYVE1 cells into 8 subclusters, and found that subcluster2 (“clu2”) was exclusively present in tumor tissues (Figure 4C). We then identified 173 “Subtype Markers” highly expressed in this cluster relative to other Macro_LYVE1 subclusters, which exhibited significant enrichment in the “IL-18 signaling pathway”, awaiting further biological investigation (Figure 4D).

## Conclusion

One of the key strengths of PreDigs lies in its comprehensive coverage of digestive tissue types and the standardized cell ontology tree, offering unique features compared to existing resources. Each cell type node is accompanied by extensive marker calculations, which we expect to facilitate single-cell annotation across various research contexts, particularly in cross-tissue comparisons to explore cellular heterogeneity and similarities among digestive organs.

However, the construction of this cell ontology tree currently relies heavily on available marker annotations. The initial version of PreDigs focuses on gastrointestinal cancers, given the challenges of constructing a new cell type hierarchy and the substantial effort required for large-scale data integration. Moving forward, we aim to extend PreDigs to encompass a broader range of cell and tissue types, particularly by integrating datasets for digestive tumor subtypes not yet represented. To further enhance the database’s utility, we plan to incorporate multi-omics data, such as spatial transcriptomics and single-cell T cell receptor sequencing. In addition, we will introduce large language models to improve single-cell classification by leveraging their ability to process complex marker-based annotations and cross-dataset comparisons.

Combined with annual data updates, these advancements will ensure that PreDigs continues to evolve as a comprehensive resource for digestive system research.

## Method

### Dataset collection and pre-processing

A total of 99 original scRNA-seq datasets for the adult digestive system were collected from 7 major public data resources as of February 2024, including National Center for Biotechnology Information (NCBI) Gene Expression Omnibus (GEO), Human Cell Landscape (HCL) [22], Human Cell Atlas (HCA), ArrayExpress, Genome Sequence Archive for Human (GSA-Human) [23], Gut Cell Survey, and Blueprint. These datasets originate from various sequencing platforms, including 10X Genomics, Microwell, Smart-seq2, Drop-seq, STRT-seq, NovaSeq, and Fluidigm. The gene expression matrices, as well as the metadata tables containing cell type labels (if accessible in publications), were downloaded. By manually reviewing each publication and its supplemental materials, we curated the metadata for each dataset, including participant, cell number, cell type number, cancer type, technology, and dataset sources. The curated metadata was elaborated in the database.

Prior to standardized pre-processing, these 99 datasets were subdivided into 124 independent data subsets categorized by three tissue types: normal tissue, paracancerous tissue, and tumor tissue. All the scRNA-seq data were processed using R (v3.6.1) with uniform methods except special explanation. Doublets were removed using DoubletFinder (v2.2.0) [24] (7% per 10000 cells) for all datasets. Seurat (v2.3.2) [25] was employed to perform a series of analyses: quality control [200 < nfeature < 5000 and mitochondrial (MT) gene percentage < 10%], normalization, principal component analysis (PCA), dimensionality reduction, cell clustering with the top 30 principal components, UMAP visualization, and differential gene expression analysis through the “FindAllMarkers” function with Wilcoxon rank-sum test (adjusted *P* value < 1E−04, |log_2_ fold change| > 1.5). For datasets generated by full-length sequencing, transcript per million (TPM) normalization was mainly performed instead of the Seurat workflow.

Batch removal and data integration were performed by Harmony (v1.0) [26] and canonical correlation analysis algorithm in Seurat (v2.3.2) [25]. To integrate the data, we first identified variable genes in each dataset with the “FindVariableFeatures” function. We then scaled the data using the “ScaleData” function and proceeded to integrate different datasets using Seurat’s “FindIntegrationAnchors” and “IntegrateData” functions, with parameters set as follows: k.filter = 50, resolution = 0.5, and dims = 30. The aforementioned processes were facilitated by Visualization integrated Precision Medicine Analytics Platform (ViPMAP; https://www.biosino.org/vipmap/).

### Cell type annotation and clustering

We performed unified cell type annotations on the pre-processed gene expression matrix. The annotation process for healthy gastrointestinal tissues mainly consisted of three steps. First, we used the tool CellAssign [27] for automated cell type prediction. Next, manual annotation was conducted based on the expression of typical cell markers. Then, cell labels were determined by comparing the re-annotated results with the original literature. Finally, cell label names were manually curated following the standardized terms in the CL database. For malignant cell classification in tumor tissues, we further refined the annotation by incorporating the expression of typical tumor cell marker genes (*EPCAM*, *TP53*, *etc.*).

Furthermore, to enhance cell type resolution, we conducted additional subtype annotations and classifications for six major cell types, including T cells, natural killer cells, myeloid cells, endothelial cells, B cells, and fibroblasts, using sophisticated reference data [6,28–32] (Table S1). For the first four cell types, larger-scale single-cell atlases were used as the reference dataset, combined with SingleR [33] and scibetR [34] for automatic refinement. Then, the re-annotated results were compared with the cell labels in the original literature. For the latter two cell types, due to the lack of available large-scale reference atlases, typical subtype markers were mainly referenced, combined with Semi-supervised Category Identification and Assignment (SCINA) [35] for automatic annotation. Similarly, the re-annotated results were compared with the labels in the original literature.

Additionally, cross-tissue data integration and clustering were utilized to analyze cell subtypes in the TME. First, single-cell data from various organs were integrated using Seurat (v2.3.2) [25] to classify and analyze cell subtypes, leading to the identification of tumor-specific subclusters. Secondly, Seurat’s canonical correlation analysis algorithm was applied to combine and cluster multiple datasets within the same gastrointestinal tumor, allowing differences in gene expression patterns of the same cell type across normal, paracancerous, and tumor tissues to be examined.

### Cell ontology tree construction

Following the comprehensive annotation of digestive single cells, a standardized ontology for cell types was constructed to structurally describe these cell types in the scRNA-seq dataset of PreDigs. This cell ontology tree was manually curated and derived from the CL reference database. A complete list of digestive cell types was obtained and matched to the existing CL hierarchy using standardized nomenclature. Subsequently, the corresponding CL numbers and parent node cell types for each cell type were compiled and organized. A unified and structured cell ontology tree was then constructed, mainly categorizing cells into five groups: immune cells, stromal cells, epithelial cells, secretory cells, and others. The text descriptions corresponding to these cell types were also documented and uniformly stored in the database.

To construct a comprehensive cell type marker set for human digestive organs and accurately profile digestive cellular characteristics, manual curation was performed based on existing resources, such as CellMarker 2.0 [13] and CellMatch [16], and canonical markers that had been experimentally validated were collected.

### Approach for deriving three types of context-specific cell markers

To uncover expression differences of the same cell type across different tissue types, the Seurat package was employed to conduct three distinct differential gene expression analyses for specific cell types under various conditions, including “Cell Markers”, “Subtype Markers”, and “TPN Markers”. First, significantly up-regulated DEGs in the specific cell type, compared to other cell types, were identified within each tailored dataset (“Cell Markers”). The heterogeneity of cell type-specific DEGs across different tissues was then elucidated by comparing these DEG lists. Next, all datasets across digestive organs containing the specific cell type were integrated to construct a large-scale cell integration dataset. Expression differences between subtypes within the same cell type were analyzed using this integrated dataset (“Subtype Markers”). Finally, to meet the research needs specific to a particular digestive organ, relevant datasets were consolidated, and an in-depth analysis of the gene expression differences for the specific cell type across different tissue types (normal tissue, paracancerous tissue, and tumor tissue) was carried out after eliminating batch effects (“TPN Markers”).

### Data visualization and database construction

In addition to basic visualization of cell type composition statistics and DEG analysis, the UMAPPlot and FeaturePlot functions in Seurat (v2.3.2) [25] were employed for cell clustering visualization and feature expression. Cell–cell interaction networks were constructed by CellChat [36]. PPI networks were constructed by STRINGdb [37]. KEGG enrichment analysis on DEGs was performed for each specific cell type using the R package clusterProfiler [38]. Survival analysis was performed by the R package survival. Static figures in the database were created by ggplot2.

PreDigs web portal was constructed by standard database development techniques. Hyper Text Markup Language 5 (HTML5) and Cascading Style Sheets (CSS) were used for front-end page display and My Structured Query Language (MySQL) was served as a container for back-end data storage. ECharts and Highcharts were adopted for building interactive graphs. The following browsers were recommended for better compatibility: Google Chrome, Firefox (v64.0 and up), or Internet Explorer (v11.0 and up). The whole bioinformatic analysis results in PreDigs were generated by in-house R scripts.

## Code availability

PreDigsR, a standalone version of the PreDigs database for cell type annotation, is available on GitHub (https://github.com/BioMedBigDataCenter/predigsr). It has also been submitted to BioCode at the National Genomics Data Center (NGDC), China National Center for Bioinformation (CNCB) (BioCode: BT007624), which is publicly accessible at https://ngdc.cncb.ac.cn/biocode/tool/7624.

## Supplementary Material

qzaf066_Supplementary_Data

## Data Availability

All data in PreDigs are freely available at https://www.biosino.org/predigs/, with no login or registration required. The database has been submitted to Database Commons [39] at the NGDC, CNCB, which is publicly accessible at https://ngdc.cncb.ac.cn/databasecommons/database/id/9719.

## CRediT author statement

**Jiayue Meng:** Investigation, Formal analysis, Data curation, Writing – original draft, Writing – review & editing. **Mengyao Han:** Data curation. **Yuwei Huang:** Resources. **Liang Li:** Investigation. **Yuanhu Ju:** Software. **Daqing Lv:** Software. **Xiaoyi Chen:** Investigation. **Liyun Yuan:** Projection administration, Methodology, Writing – original draft, Writing – review & editing. **Guoqing Zhang:** Conceptualization, Projection administration, Funding acquisition, Writing – original draft, Writing – review & editing. All authors have read and approved the final manuscript.
