# Supplementary material for: PreDigs: A Database of Context-specific Cell Type Markers and Precise Cell Subtypes for Digestive Cell Annotation
Source: Genomics Proteomics Bioinformatics. 2025 Aug 7;23(4):qzaf066. doi: 10.1093/gpbjnl/qzaf066 (PMC12571502; doi:10.1093/gpbjnl/qzaf066)
Supplement: qzaf066_Supplementary_Data [file qzaf066_supplementary_data.zip › FigureS2.pdf]

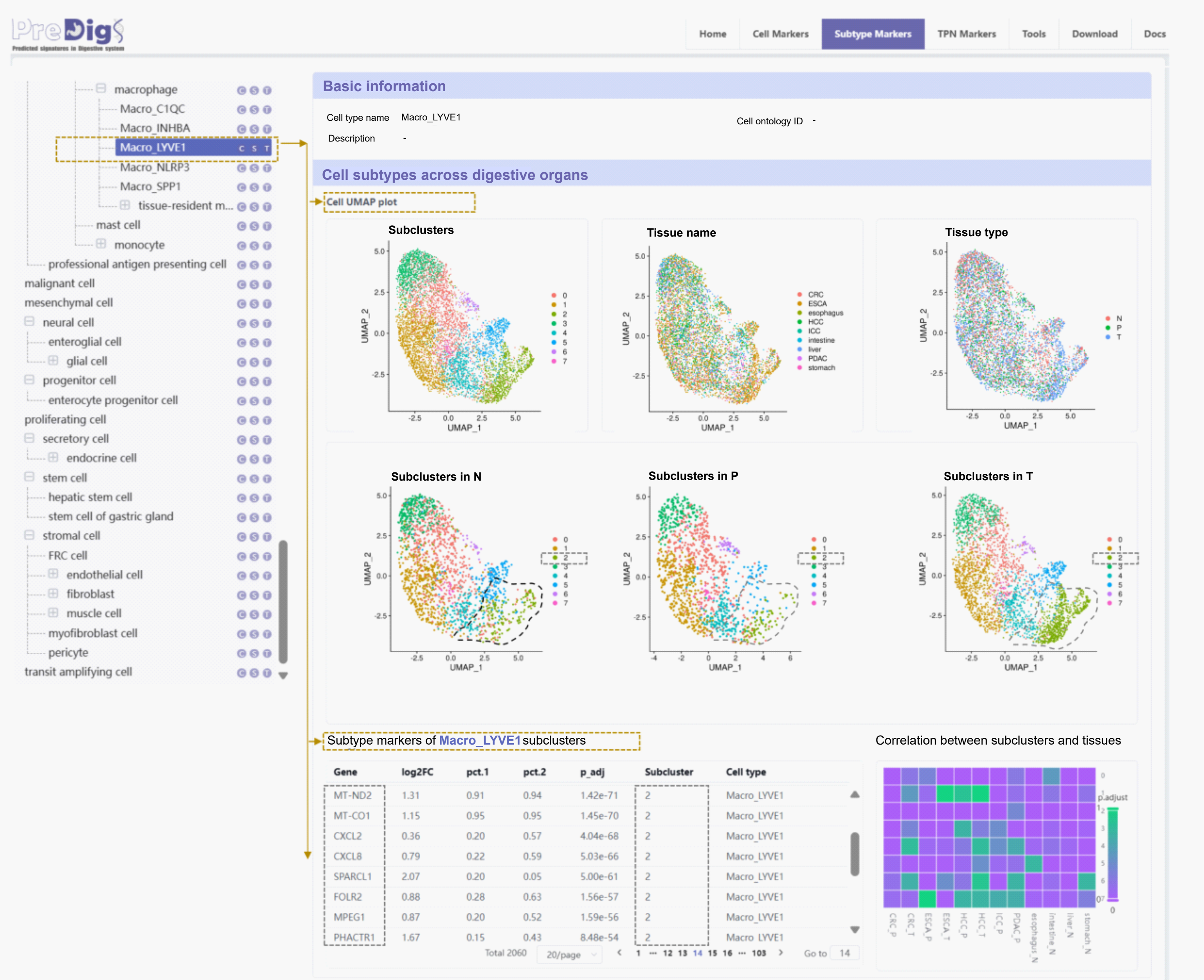
PreDigs
Predicted signatures in Digestive system
Home
Cell Markers
Subtype Markers
TPN Markers
Tools
Download
Docs
macrophage
Macro_C1QC
Macro_INHBA
Macro_LYVE1
Macro_NLRP3
Macro_SPP1
tissue-resident m...
mast cell
monocyte
professional antigen presenting cell
malignant cell
mesenchymal cell
neural cell
enteroglial cell
glial cell
progenitor cell
enterocyte progenitor cell
proliferating cell
secretory cell
endocrine cell
stem cell
hepatic stem cell
stem cell of gastric gland
stromal cell
FRC cell
endothelial cell
fibroblast
muscle cell
myofibroblast cell
pericyte
transit amplifying cell
Basic information
Cell type name Macro_LYVE1
Cell ontology ID -
Description -
Cell subtypes across digestive organs
Cell UMAP plot
Subclusters
Tissue name
Tissue type
CRC
ESCA
esophagus
HCC
ICC
intestine
liver
PDAC
stomach
N
P
T
UMAP_1
UMAP_2
Subclusters in N
Subclusters in P
Subclusters in T
Subtype markers of Macro_LYVE1 subclusters
Correlation between subclusters and tissues
Gene | log2FC | pct.1 | pct.2 | p_adj | Subcluster | Cell type
MT-ND2 | 1.31 | 0.91 | 0.94 | 1.42e-71 | 2 | Macro_LYVE1
MT-CO1 | 1.15 | 0.95 | 0.95 | 1.45e-70 | 2 | Macro_LYVE1
CXCL2 | 0.36 | 0.20 | 0.57 | 4.04e-68 | 2 | Macro_LYVE1
CXCL8 | 0.79 | 0.22 | 0.59 | 5.03e-66 | 2 | Macro_LYVE1
SPARCL1 | 2.07 | 0.20 | 0.05 | 5.00e-61 | 2 | Macro_LYVE1
FOLR2 | 0.88 | 0.28 | 0.63 | 1.56e-57 | 2 | Macro_LYVE1
MPEG1 | 0.87 | 0.20 | 0.52 | 1.59e-56 | 2 | Macro_LYVE1
PHACTR1 | 1.67 | 0.15 | 0.43 | 8.48e-54 | 2 | Macro_LYVE1
Total 2060
20/page
1 ... 12 13 14 15 16 ... 103
Go to 14
p.adjust
CRC_P
CRC_T
ESCA_P
ESCA_T
HCC_P
HCC_T
ICC_P
PDAC_P
esophagus_N
intestine_N
liver_N
stomach_N
