# Supplementary material for: PreDigs: A Database of Context-specific Cell Type Markers and Precise Cell Subtypes for Digestive Cell Annotation
Source: Genomics Proteomics Bioinformatics. 2025 Aug 7;23(4):qzaf066. doi: 10.1093/gpbjnl/qzaf066 (PMC12571502; doi:10.1093/gpbjnl/qzaf066)
Supplement: qzaf066_Supplementary_Data [file qzaf066_supplementary_data.zip › Supplementary material captions.docx]

# Supplementary material

**Figure S1 Data processing and construction workflow of PreDigs**

The workflow of the PreDigs database and platform focuses on cell type taxonomy and context-specific markers. In the first step, data collection and preprocessing involve gathering scRNA-seq datasets from organs such as the pancreas and liver. Next, cell type annotation and clustering are performed, including quality control, normalization, and clustering to ensure accurate annotation. Subsequently, context-specific markers are calculated, identifying DEGs and subtype markers across 124 datasets. Following this, an 8-level cell ontology tree is constructed, and three types of context-specific markers are curated. Then, an online visualization platform is provided, incorporating tools such as scibetR and SCINA. Finally, the database and platform are deployed on Docker Hub and a local platform, offering a user-friendly interface. CL, Cell Ontology; QC, quality control.

**Figure S2 The screenshot of the “Subtype Markers” page in PreDigs**

The page provides a comprehensive analysis of digestive system cell subtypes, focusing on their distribution and marker expression across tissues. Users can explore refined clustering through the left cell ontology tree, examine subtype distributions across different tissues and tissue types, and access detailed marker lists for each subcluster. By integrating these features, the platform enables precise identification of cell subtypes and their markers, supporting research on cell heterogeneity, and functional diversity within digestive tissues.

**Figure S3**  **The screenshot of the “TPN Markers” page in PreDigs**

This page presents a comparative analysis of markers for the same cell type across different tissue types. The left panel shows the cell ontology tree for selection, while the upper section allows tissue switching. The distribution and proportion of the selected cell type are displayed alongside statistical analyses. A comprehensive marker table is provided, with visualizations of expression plots, protein–protein interactions, survival analysis, and KEGG enrichment analysis illustrating functional associations.

**Table S1 Cell subtype annotation strategies and reference data types**
