# Supplementary material for: PreDigs: A Database of Context-specific Cell Type Markers and Precise Cell Subtypes for Digestive Cell Annotation
Source: Genomics Proteomics Bioinformatics. 2025 Aug 7;23(4):qzaf066. doi: 10.1093/gpbjnl/qzaf066 (PMC12571502; doi:10.1093/gpbjnl/qzaf066)
Supplement: qzaf066_Supplementary_Data [file qzaf066_supplementary_data.zip › Table S1.docx]

**Table S1 Cell subtype annotation strategies and reference data types**

| **Cell type** | **Cell subtype** | **Reference data type** | **Ref.** |
| --- | --- | --- | --- |
| T cell | Teff, Th17, Tfh, CTL, Treg, Tem, Tcm, Tn, Tex, NKT, Gamma-delta T... | Gene expression matrix | [27] |
| Natural killer cell | CD56^bright^CD^low^NK, CD56^dim^CD16^hi^NK | Gene expression matrix | [28] |
| Myeloid leukocyte | cDC, Neutrophil, Mast cell, Classical monocyte, Non-classical monocyte | Gene expression matrix | [6] |
| Endothelial cell | Lymphatic, Artery, Vein, Tip cell, TEC, Stalk cell, Capilary | Gene expression matrix | [31] |
| B cell | Plasma, Naive B, Memory B | Canonical markers for  Plasma cell: *SDC1*, *MZB1*, *IGHG1*, *IGHA1*, *IGKC*  Naive B cell: *MS4A1*, *IGHD*, *FCER2*, *TCL1A*, *IL4R*  Memory B cell: *MS4A1*, *CD27*, *AIM2*, *TNFRSF13B* | [29] |
| Fibroblast | myofibroblastic CAF (myoCAF), immune regulatory& inflamatory (iCAF) | Canonical markers for  myoCAF: *COL1A1*, *COL10A1*, *COL4A1*, *MMP3*, *IL4*, *IL13*, *TGFB1*, *ACTG2*, *ACTA2*, *FAP*, *PDPN*  iCAF: *IL6*, *IL11*, *IL8*, *LIF*, *CSF2*, *CXCL1*, *CXCL12*, *CXCL14*, *CCL2*, *CCL8*, *CFD*, *C1QC*, *C1QA*, *C1QC*, *HLA-DRA* | [30] |

*Note*: For T cells, natural killer cells, myeloid cells, and endothelial cells, we used large-scale pan-cancer atlases as the reference dataset, combined with SingleR and scibetR tools for automatic fine annotation. For B cells and fibroblasts, canonical subtype markers were mainly used alongside SCINA for automatic annotation.
