# Supplementary figures and images for: PreDigs: A Database of Context-specific Cell Type Markers and Precise Cell Subtypes for Digestive Cell Annotation
Source: Genomics Proteomics Bioinformatics. 2025 Aug 7;23(4):qzaf066. doi: 10.1093/gpbjnl/qzaf066 (PMC12571502; doi:10.1093/gpbjnl/qzaf066)

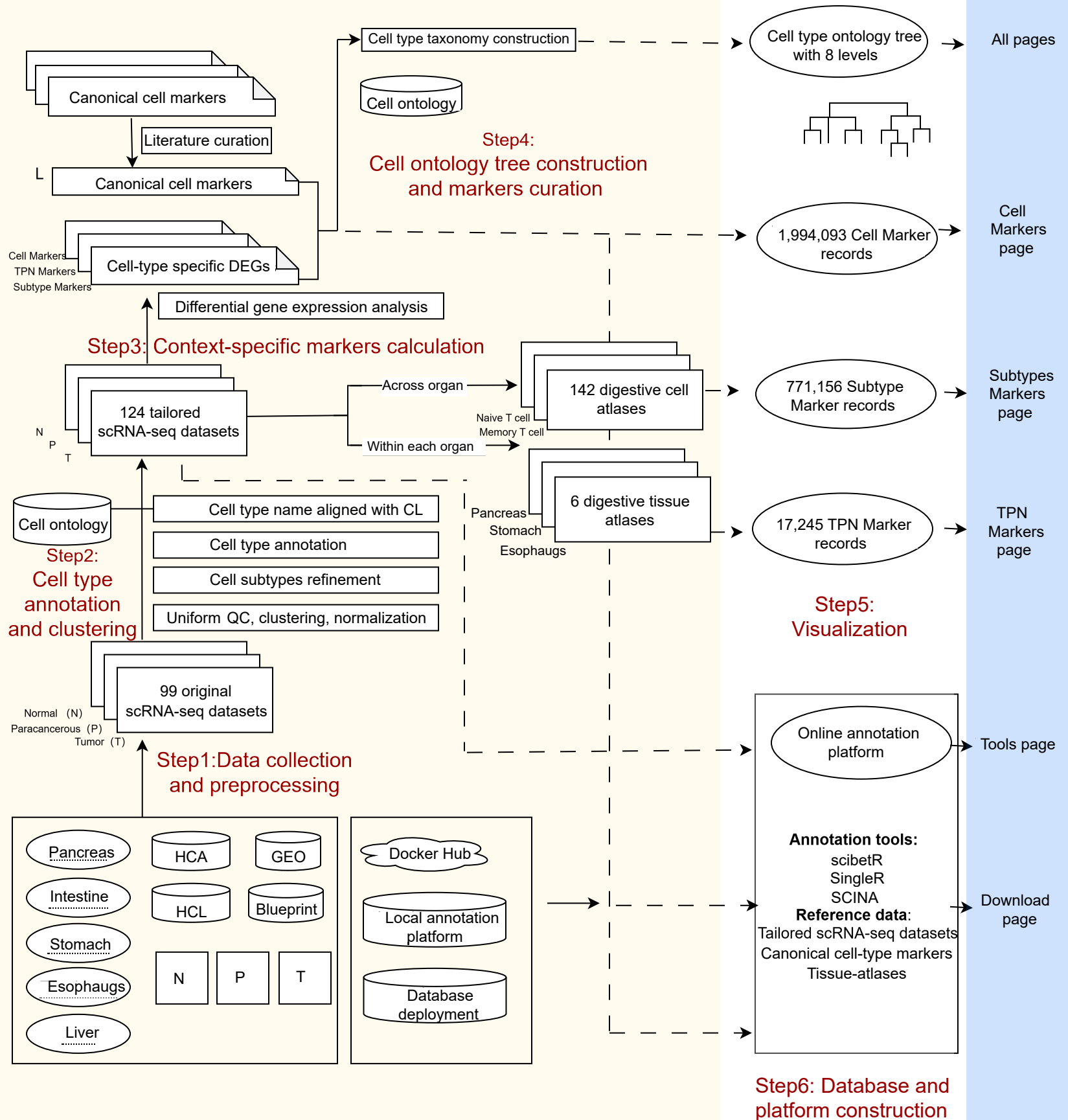

Data processing workflow in PreDigs

PreDigs data

Interface

Supplement: qzaf066_Supplementary_Data [file qzaf066_supplementary_data.zip › FigureS1.pdf]

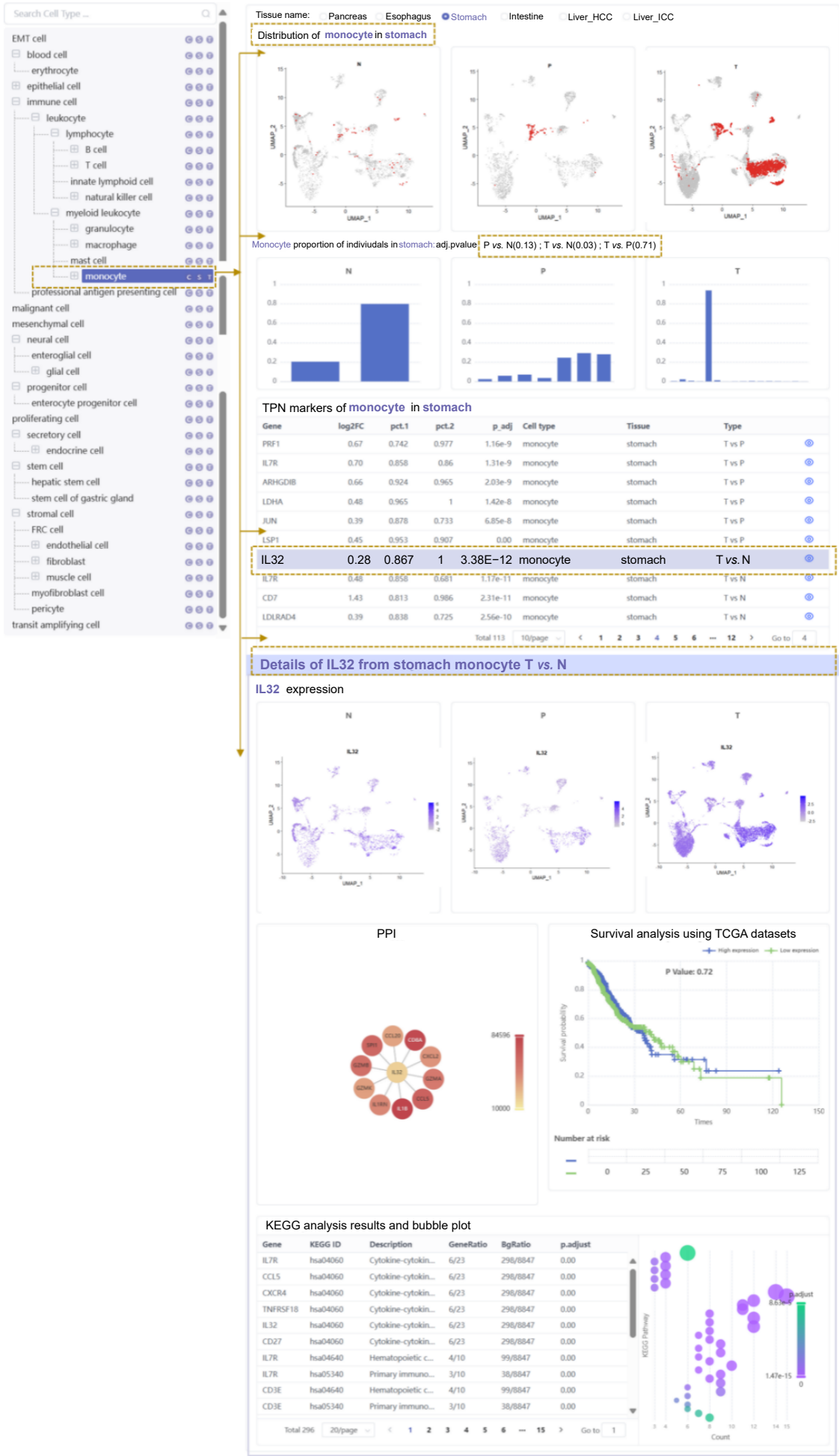

Supplement: qzaf066_Supplementary_Data [file qzaf066_supplementary_data.zip › FigureS3.pdf]
